# Disaster management education in environmental health programs: Academic perspectives within the South African higher education context

**DOI:** 10.4102/jamba.v17i1.1949

**Published:** 2025-10-25

**Authors:** Patience Mbola, Davies V. Nkosi, Oyewale M. Morakinyo

**Affiliations:** 1 Department of Environmental Health, Faculty of Science, Tshwane University of Technology, Pretoria, South Africa; 2 Department of Environmental and Occupational Studies, Faculty of Applied Science, Cape Peninsula University of Technology, Cape Town, South Africa; 3 Department of Environmental Health Sciences, Faculty of Public Health, University of Ibadan, Ibadan, Nigeria

**Keywords:** disaster management, emergencies, education, environmental health, competencies, disasters

## Abstract

Environmental health practitioners (EHPs) are expected to possess critical competencies in disaster risk reduction, emergency response and recovery, which should ideally be developed during undergraduate education. However, since the transition in 2016 from diploma to degree programmes in environmental health in South African higher institutions, there has been limited insight into the adequacy of disaster management training in these programmes. This study aimed to analyse the training content, course structures and competency frameworks currently employed to prepare EHPs in South Africa for roles in disaster management. An exploratory concurrent mixed-methods design examined the current structure and delivery of disaster management modules in environmental health programmes across South Africa. A semi-structured self-administered questionnaire was used to elicit information on the relevance of disaster management in environmental health, course delivery, competency approaches and work-integrated learning. Findings revealed systemic inconsistencies and critical gaps in curriculum implementation. These challenges included the lack of module duration, structure standardisation and the limited integration of work-integrated learning and digital learning methodologies. The study concludes that a standardised national curriculum is needed that is aligned with the South African Disaster Management Framework, the EHP professional scope of practice and the Sendai Framework for Disaster Risk Reduction.

**Contribution:**

The study recommends establishing a Disaster Management Academics Forum to improve curriculum consistency, encourage academic collaboration and promote ongoing quality enhancement. These measures are essential for maintaining uniform graduate skills, which will strengthen the professional capacity of EHPs as frontline workers in disaster risk reduction and further reinforce South Africa’s long-term resilience.

## Introduction

In response to the Hyogo Framework for Action (2005–2015), which emphasised the importance of disaster risk reduction through education, awareness and capacity-building, the African Academy for Environmental Health (AAEH) introduced a harmonised curriculum for environmental health (EH) degree programmes in 2010 (Mbola, Nkosi & Morakinyo 2024; The African Academy for Environmental Health 2010; UNISDR 2005). This curriculum included a disaster management module intended for adoption by African institutions offering EH training. The integration of this module emphasised the need for adequately trained academic staff capable of preparing this new generation of environmental health practitioners (EHPs), equipped to respond effectively to disasters (Berhanu et al. 2016; Mbola et al. 2024). This development paralleled broader global and regional advocacy for the inclusion of disaster education in health professional training programmes, particularly considering the persistent gaps in knowledge and skills among various health professionals, including EHPs, regarding disaster preparedness and response (Achora & Kamanyire 2016; Langan et al. 2017; Mbola et al. 2024; Ning et al. 2014; Williams, Nocera & Casteel 2008). Environmental health practitioners are often among the frontline responders during public health emergencies, tasked with assessing disaster impacts and evaluating response interventions at the community level (Rodrigues et al. 2021). Their community-oriented scope of practice and multidisciplinary training position them as key contributors to disaster management efforts.

Consequently, it is expected that EHPs possess critical competencies in disaster risk reduction, emergency response and recovery (Mbola et al. 2024; Reischl, Sarigiannis & Tilden 2008; Rodrigues et al. 2021; Ryan 2018). These competencies should ideally be learned during undergraduate training, work-integrated learning and community service placements. Despite some progress in integrating disaster-related competencies within EH education, concerns persist regarding the standardisation of disaster education across institutions (Hung et al. 2021). Variations may exist in course structures, competency frameworks and terminology used in different universities (Hung et al. 2021). Evidence of these gaps in EH training can be seen in a study conducted in Kenya, which found that while EH graduates were generally willing to engage in public health emergency responses, they reported insufficient training in disaster management (Jepngetich et al. 2018). Similarly, in Uganda, EHPs were found to lack core competencies in disaster preparedness and infection prevention and control (Walekhwa, Kizza & Musoke 2022). Research from Nigeria also identified disaster prevention and preparedness as key challenges within the EH profession (Joshua et al. 2023). Moreover, Berhanu et al. (2016) observed that many health professionals, including EHPs, demonstrated limited knowledge of disaster response and lacked opportunities for continuous professional development in disaster management. These findings highlight the importance of solid foundational disaster management education in enhancing EHPs’ capacity to mitigate health risks and manage emergencies effectively. While structured undergraduate training should provide this essential base, it should also be complemented by continuous professional development initiatives in line with the EH scope of practice (Mbola et al. 2024; South Africa 2009). Strengthening the training this way is crucial to produce an EHP that is competent, responsive and adaptable to the diverse disaster-prone contexts across South Africa and the broader African region. This study aims to analyse the training content, course structures and competency frameworks currently utilised to prepare EHPs in South Africa for disaster management roles.

## Research methods and design

### Study design

The study employed a mixed-methods approach with an exploratory, concurrent design to examine the current structure and delivery of disaster management modules within EH programmes in South Africa. The concurrent design facilitated the simultaneous collection and analysis of quantitative and qualitative data, enabling researchers to obtain a comprehensive understanding of the phenomenon (Tashakkori & Teddlie 2010).

### Study population

The study population consisted of disaster management experts affiliated with accredited institutions that offered EH training in South Africa. A purposive sampling strategy was utilised to identify suitable participants with relevant expertise. In addition, snowball sampling was utilised to expand the participant pool, whereby known trainers were asked to forward the study invitation to other eligible experts.

Alternative sampling methods such as random sampling and stratified sampling were considered; however, they were deemed unsuitable for this study. Random sampling would not have ensured the inclusion of participants with specialised knowledge of disaster management and EH training, which was key to addressing the study objectives (Etikan, Musa & Alkassim 2016). Likewise, stratified sampling would have needed a comprehensive sampling frame of all disaster management and EH experts in South Africa, which is readily available (Iliyasu & Etikan 2021). Purposive sampling, by distinction, enabled the deliberate selection of participants with the required expertise, while snowball sampling facilitated access to additional experts in this relatively small and specialised professional group (Etikan et al. 2016).

### Development of a data collection tool

The development of the questionnaire was informed by current scientific literature, relevant legislation and professional standards guiding EH practice. Key reference materials included the environmental health curriculum developed by the African Academy for Environmental Health (2010) (The African Academy for Environmental Health 2010); the World Health Organization’s (WHO) practical guide, Environmental Health in Emergencies (Wisner & Adams 2002); the Scope of Practice for Environmental Health in South Africa; and the Sphere Handbook: Humanitarian Charter and Minimum Standards in Humanitarian Response (WHO 2018). These reference sources were supplemented by an extensive literature review of existing research on emergency preparedness among public health professionals, ensuring that the questionnaire aligns with both international and national perspectives on disaster risk management within the EH context.

The final questionnaire consisted of sixty-one items, structured into four key sections to capture comprehensive data relevant to the study objectives: (1) biographical information of participants, (2) disaster management in relation to environmental health practices, (3) educational programmes focusing on the teaching and training of disaster management in EH curricula and (4) work-integrated learning (WIL) or experiential learning components linked to disaster management education. This approach allowed for the collection of both descriptive and experiential data, contributing to a holistic understanding of disaster management education within EH training institutions in South Africa.

### Data collection and analysis

Data were collected using a self-administered, semi-structured questionnaire, following ethical clearance granted by the Tshwane University of Technology Ethics Committee (REC2023-12-087) in March 2024. Each participant received an email with an invitation letter, an information sheet and an informed consent form. Participation was voluntary, and all ten participants completed and returned the signed consent forms along with the completed questionnaires.

Given the exploratory nature of the study and the relatively small sample size (*N* = 10), a concurrent mixed-methods design was adopted. This design is aligned with the convergent approach in mixed-methods research, where qualitative and quantitative data are collected and analysed in parallel to complementary insights (Creswell & Clark 2017). The quantitative data derived from the structured items in the questionnaire were analysed descriptively using frequency counts and percentages. Results are presented in tables and figures to highlight key patterns and institutional trends.

The qualitative data were extracted from the open-ended responses to the questionnaire. These responses were then thematically analysed to identify, interpret and clarify emerging patterns and themes relevant to the study objectives (Koen et al. 2024). The analysis followed the six-phase process outlined by Koen et al. (2024):

- Familiarisation with the data by repeatedly reading participants’ responses.
- Generating initial codes by identifying significant phrases and ideas.
- Searching for themes by grouping codes into meaningful categories.
- Reviewing themes to ensure they align with the coded data.
- Defining and naming themes to capture the essence of each category.
- Producing the report by synthesising the themes about the study objectives.

The qualitative findings were further organised into tables and figures to ensure there is clarity and to facilitate integration with the quantitative data. The integration of both quantitative and qualitative methods occurred during the interpretation phase to allow for a deep understanding of how disaster management education is currently approached in South African EH training institutions. Findings are presented in the ‘Results’ section.

### Ethical considerations

Ethical clearance to conduct this study was obtained from Tshwane University of Technology Research Ethics Committee (Ref: REC. 2023-12-087).

## Results

### Demographics

The demographic characteristics of the participants are summarised in Table 1. As expected for academic staff affiliated with institutions of higher learning, nine participants held a minimum qualification of National Qualification Framework (NQF) Level 9 (master’s degree), with three of these holding a Level 10 qualification (Doctorate). In addition, two participants reported that they were currently pursuing doctoral studies. Most participants (8 out of 10) held professional qualifications in EH and were registered as Independent EHPs with the Health Professions Council of South Africa (HPCSA). Their practical field experience ranged from 1 year to 14 years. All respondents were affiliated with one of the seven institutions of higher learning accredited by the HPCSA to offer EH degrees. Furthermore, two participants were employed full-time in municipal disaster risk management departments that collaborate with universities to facilitate Work-Integrated Learning (WIL). One participant was employed part-time as a disaster management lecturer while concurrently working as an EHP within a municipal setting.

**TABLE 1:** Demographics of disaster management experts involved in environmental health training who completed the questionnaire.

Demographics	No. of Participants
**Highest education level**	
Doctorate/PhD	3
Masters	6
Bachelor’s degree	1
**Professional degree/discipline**	
Environmental health	8
Natural science	1
Disaster risk management	1
**Number of years of professional experience as an EHP**	
0	3
1–5	4
6–10	2
11–14	1
**Current employment category or work environment**	
Academic institution (with one also working in a municipality as an EHP)	8
Disaster Risk Management Department (Municipality)	1
Disaster Management Training Department (Municipality)	1
**Position as a disaster management expert in environmental health**	
Academic lecturer	8
Disaster management facilitator (WIL)	1
Guest/Academic lecturer (WIL)	1
**Nature of employment contract as a disaster management expert**	
Full-time (academic lecturer)	6
Part-time (academic lecturer)	1
Contract academic lecturer (research fellow)	1
Full-time (disaster management practitioner)	2
**Number of years in the employment contract (in the current academic institution/municipality)**	
1–5	2
6–10	4
11–15	1
16–20	3
**Types of academic institutions working in/with as disaster management experts in EH**	
University	2
University of Technology	6
Other (municipal disaster departments)	2
**Any form of disaster management-related training/short courses that the expert has acquired or attended?**	
Yes	7
No	3
**Disaster management training background**	
Short-course programmes	2
Continuous professional development training	2
Undergraduate and postgraduate courses	2
Self-learning and experience	4

EHP, environmental health practitioners; EH, environmental health; WIL, work-integrated learning.

In terms of disaster management training, seven participants reported having completed at least one form of disaster management-related education or training. These included formal qualifications, continuing professional development (CPD) programmes and self-directed learning through professional practice. These findings highlight the diverse expertise and training pathways among disaster management experts involved with training in the environmental health field.

The following section presents participants’ views on the design, structure and overall implementation of the disaster management module within environmental health training programmes.

### Disaster management module structure

#### Purpose of the disaster management module in relation to the environmental health profession

Three themes were generated based on the responses of the participants regarding the purpose of disaster management in environmental health, namely: (1) disaster preparedness, response and risk reduction; (2) the role of EHPs in disaster situations; and (3) climate change and the need for disaster management.

#### Disaster preparedness, response and risk reduction

Eight of the ten participants highlighted that the disaster management module is designed to equip students and EHPs with the necessary skills to prepare for, respond to, and recover from disasters, thereby minimising their health and environmental impacts. One participant emphasised:

‘I believe the purpose is to prepare individuals to anticipate, respond to, and recover from disasters that could directly or indirectly impact environmental conditions and public health.’ (Participant 10, Disaster Management Facilitator, Miss [Masters])

The module also focuses on core principles of disaster management, including risk assessment, mitigation, emergency response and the restoration of essential services such as clean water and sanitation. These foundational concepts ensure that students develop a comprehensive understanding of disaster management in the context of environmental health.

#### Important role of environmental health practitioners in disaster situations

Five of the 10 participants elaborated that the module clarifies the expected roles and responsibilities of EHPs in disaster events, ensuring they are adequately prepared to function in multidisciplinary teams during disaster response and management. By integrating this training into the curriculum, students gain competencies that enable them to contribute effectively to disaster management efforts.

#### Climate change and the need for disaster management

One of the ten participants cited the increasing impact of climate change, such as extreme weather events like floods and droughts, as a key reason for integrating disaster management into the environmental health curriculum. The participant acknowledged the critical role of EHPs in preventing further outbreaks and disasters. The participant stated:

‘Climate change has necessitated the inclusion of disaster management in the EH curriculum, recognising that EHPs play a critical role in preventing further outbreaks and disasters.’ (Participant 3, Academic lecturer, Professor)

### Disaster management curriculum

#### Structure and implementation of disaster management module

All participants (*N* = 10) confirmed that the disaster management module is offered at the fourth-year level of the Bachelor of Environmental Health programme, where it is a compulsory component. The duration of the module varied across institutions. Four participants indicated that the module is offered as a semester-long course, while five reported that it is structured as a year-long module. One participant was uncertain about the duration. Regarding prerequisite requirements, five participants noted that although the module titles may differ between institutions, several core modules, such as food quality and safety, epidemiology, management practice, occupational health and safety and environmental pollution, are generally considered key and should ideally be prerequisites to the disaster management module. However, four participants stated that there were no formal prerequisite modules, and one was unsure about whether any specific modules should be completed in advance.

Participants were also asked to indicate the number of students enrolled in the disaster management module for the 2024 academic year. Reported enrolment figures varied considerably across institutions. Of the 10 participants, 5 provided specific numbers: 2 reported 32 students each, 2 reported 22 students each, 1 indicated 40 students and another reported 65 students enrolled. One participant mentioned having less than 20 students registered. The remaining three participants did not provide numerical data, citing reasons such as uncertainty or not currently teaching the fourth-year cohort in 2024.

### Key content in disaster management curriculum

An evaluation of the study units (key content areas) reported by participants revealed both common themes and some variability in the disaster management module across different higher education institutions. In response to an open-ended question regarding the content areas covered in the module, three out of ten (*N* = 10) participants provided detailed descriptions of the content, three offered general topic headings and four left the section blank. One participant clarified that the module is co-lectured at their institution, with their contribution focusing specifically on technical areas such as food safety, radiation and the management or handling of the dead on WHO guidelines. Despite differences in the depth of responses, several common content areas emerged across institutions. These included an introduction to disaster management and its basic concepts; the roles and responsibilities of EHPs during disasters; preparedness, mitigation and risk assessment; planning and institutional frameworks; and emergency response, recovery and reconstruction (as shown in Table 2). The foundational concepts were generally aimed at equipping students with an understanding of the broader context and operation of disaster management systems in alignment with their professional scope of practice. Most institutions emphasise the core responsibilities of EHPs during disaster events. These responsibilities directly align with the scope of EH practice and WHO guidelines. Table 2 provides a detailed overview of core content and competency areas.

**TABLE 2:** Core content areas and competencies in disaster management module for environmental health (*N* = 10).

Content area	Competency area	Participants
Risk assessment and management	Understand risk principles and practices	8
	Identify, evaluate and prioritise hazards	9
	Develop and implement mitigation strategies	8
Crisis communication and public information	Communicate with stakeholders during disasters	8
	Disseminate timely and accurate information	8
	Use media and communication tools effectively	8
Incident Command System (ICS)	Understand ICS structure and functions	5
	Apply ICS principles in disaster response	5
	Coordinate with other response agencies	6
Environmental health impact assessment	Assess the environmental health impacts of disasters	7
	Conduct environmental sampling and monitoring	5
	Mitigate adverse health effects	6
Epidemiology & disease surveillance	Conduct surveillance and outbreak investigations	5
	Understand the role of epidemiology in disaster response	6
	Implement public health interventions	6
Community resilience & recovery	Promote community resilience and capacity-building	8
	Develop recovery plans	6
	Engage community stakeholders	8
Legal & ethical considerations	Understand disaster-related legal frameworks	6
	Apply ethics in disaster decision-making	5
	Navigate compliance and regulation	4
Health & safety compliance	Recognise and address mental health needs	2
	Provide psychological first aid	1
	Promote mental health resilience	2
Data analysis & Interpretation	Collect, analyse and interpret disaster-related data	6
	Use data in policy and decision-making	6
	Develop data-driven management strategies	5
Public health policy & advocacy	Understand public health policy in disaster management	5
	Advocate for disaster-resilient policies	5
	Engage in policy development	5
Leadership & team coordination	Provide leadership in disaster response	3
	Coordinate multidisciplinary teams	5
	Foster collaboration in high-pressure settings	6
Technology & innovation	Use technology and innovation in disaster response	5
	Apply GIS, remote sensing and related tools	6
	Stay updated on emerging technologies	4
Confidence in students’ ability to acquire competencies	Very confident	2
	Confident	4
	Neutral	2
	Unconfident	2
Most critical competencies for EHPs	Risk assessment and analysis	2
	Crisis communication	0
	Emergency planning and coordination	0
	Community resilience building	0
	Recovery and rehabilitation	0
	All the above competencies	8

EHP, environmental health practitioners; GIS, geographic information system; ICS, Incident Command System.

Regarding student competencies reported, the highest levels were observed in areas such as risk assessment, emergency preparedness, community resilience and recovery. On the other hand, competencies related to leadership in response teams, collaborative coordination, and health and safety compliance received less emphasis across the institutions. Overall, six participants expressed confidence in their students’ ability to acquire disaster management competencies relevant to the EH profession.

The participants were also asked to identify the types of disasters forming part of the module content. Their responses indicated a variety of types of disasters incorporated into the disaster management module. Disaster types such as epidemics, flood disasters, drought disasters and earthquakes were mentioned consistently across most of the responses. It is not surprising that flood disasters were deliberately included in the content of all the responses owing to the recurrence of this disaster type in South Africa. Two participants further highlighted that their module does not include a specific type of disaster, suggesting that more types of disasters may be incorporated into their content as necessary. Another participant indicated the inclusion of other disaster types, such as man-made disasters, informal settlement fires, gale-force winds, chemical spills, gas explosions in central business districts (CBDs) and events like the Boksburg explosion.

The inclusion of these types of disasters in the curriculum of disaster management in EH somewhat suggests the extent of disaster preparedness education and comprehensiveness of the curriculum. When asked about the overall comprehensiveness of the current disaster management training programme for EH students, responses varied: five participants felt the programme was ‘somewhat comprehensive’, two believed it to be ‘very comprehensive’, while three remained neutral.

### Curriculum design and delivery of the disaster management module

Table 3 outlines key aspects essential to any module offered at an institution of higher learning, including the mode of teaching and learning. While the results indicate diverse teaching styles of disaster module packaging and offering, most institutions rely on in-person learning (*n* = 6), thus emphasising the importance of face-to-face interaction. The use of hybrid (*n* = 4) and blended learning (*n* = 4) also indicated some kind of technological integration into teaching and learning. Other teaching methods included case studies, group work and guest lectures. However, service-learning, community engagement and simulation exercises remain underutilised in the delivery of this module.

- *Blended learning*: a combination of online and face-to-face learning with online and offline learning components occurring at different times.
- *Hybrid learning*: a combination of online and face-to-face learning that occurs simultaneously, often in real time.

**TABLE 3:** Teaching and learning aspects of disaster training modules (*N* = 10).

Aspect	Response options	Number of participants
Mode of disaster course delivery	In-person	6
	Blended	4
	Hybrid	4
	Field training and simulations	3
	Workshops	1
Predominant teaching methods used	Lectures	9
	Case studies	8
	Group work	6
	Guest lectures	6
	Simulations	3
	Practical exercises	3
	Problem-based learning	3
	Service-learning	3
	Role-playing	2
	All-hazard approaches	2
	Interprofessional approach	1
	Self-study	1
Sufficient use of modern technology in teaching	Yes	1
	No	9
Inclusion of guest experts	Frequently	1
	Occasionally	6
	Rarely	3
Satisfaction with student engagement	Very satisfied	4
	Satisfied	4
	Neutral	1
	Dissatisfied	1
Evaluation methods of training effectiveness	Student feedback	9
	Performance assessments	5
	Peer reviews	4
	Self-assessment	2
	Other (module and teaching evaluations)	1
Student feedback used for course improvement	Frequently	5
	Occasionally	3
	Rarely	2
Resource accessibility level	Very accessible	6
	Accessible	4
Effectiveness of practical exercises and preparedness	Very effective	1
	Effective	7
	Neutral	2
Level of interdisciplinary integration	Very well	3
	Well	3
	Neutral	2
	Poorly	2
Areas mostly emphasised in the curriculum	All aspects (prevention, preparedness, response, recovery)	8
	Prevention and mitigation	1
	Other (identification of disasters, response as an EHP)	1
Areas needing more emphasis	Community engagement	7
	Risk assessment	6
	Emergency response planning	6
	Public health communication	4
	Recovery and rehabilitation	4
Perceived gaps in disaster training programmes	Yes	9
	No	1
Opportunities for research engagement	Yes	7
	No	2
	Missing response	1
Competency assessment methods used	Group projects	8
	Written exams/tests	7
	Practical assessments	4
	Fieldwork evaluations	3
	Continuous assessments	3
	Online simulation tests	2
	Peer reviews	1

EHP, environmental health practitioner.

**FIGURE 1:**
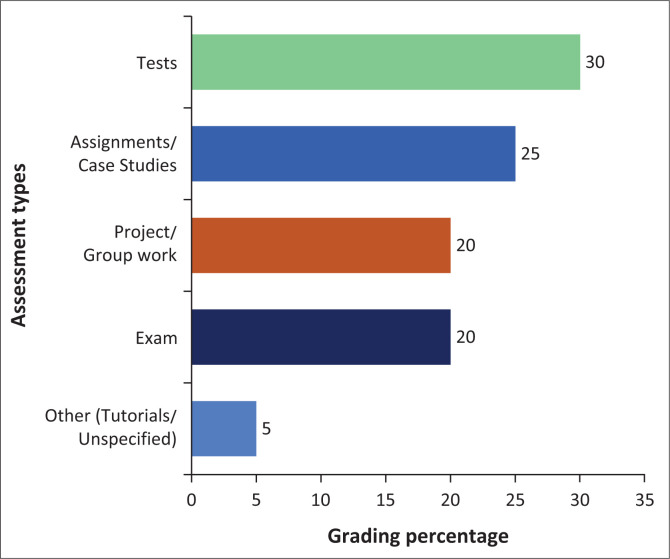
Assessment type and general grading.

Most participants (*n* = 9) reported limited use of technology in disaster management teaching, with only one institution indicating meaningful integration of technological tools into instruction. Despite this, participants expressed a high level of satisfaction with student engagement in disaster-related teaching activities. Furthermore, most institutions (*n* = 9) routinely utilise student feedback to assess the effectiveness of disaster training. However, the extent to which this feedback informs curriculum development varies. Of the ten participants, five reported frequently incorporating student input into curriculum improvement efforts, three did so occasionally and two reported rare usage. Competency levels of disaster management modules are assessed using different methods, with many institutions (*n* = 8) using group projects and written tests and/or examinations (*n* = 7) and assignments (add number). Figure 1 depicts the type of assessments and their general grading used by the seven institutions offering disaster management modules.

The assessment practices reported by participants vary in format and weighting, though they show some common patterns. Assignments and tests are the most frequently used forms of assessment. These assessment methods were interused by participants. As expected, assignments were reported by six participants (weighted at 20% – 50%) and invigilated tests by five participants, commonly weighted at 20.0% but sometimes as high as 50.0%. Some modules adopt a balanced structure, such as three assessments contributing 20%, 40% and 40%, respectively, towards the semester mark. Other forms of assessment, including creative projects (30.0%), case studies (10.0%) and hazard mapping (10.0%), were each reported once, suggesting limited but diverse practices. Group work and tutorials also appeared in a few responses, both typically contributing 10% – 20%. A few participants reported continuous assessment models contributing 100% of the semester mark, while others indicated a 50/50 split between examination and coursework. One participant mentioned averaging equal marks across two major assessments. Two participants were unclear or did not specify weighting. Overall, the data reflect a combination of traditional and alternative assessment methods with diverse implementation across institutions.

Even though all the participants accepted that disaster-related field experience is important, only 50% (*n* = 5) of them indicated that students were exposed to disaster management during WIL, which highlights the fragmentation of WIL experience in disaster management subjects. Time allocated for the disaster management module for WIL varied between 8 h and 2 days, as noted by four participants, while one participant indicated that there is no specific time allocation. Table 4 shows a summary of WIL activities related to disaster management.

**TABLE 4:** Work-integrated learning activities related to disaster management in environmental health training.

WIL theme	Response	Number of participants
Student exposure to disaster management during WIL	Yes	5
	No	2
	I do not know	3
Perceived importance of field experience in disaster management	Very important	7
	Important	3
Time allocated to disaster management during WIL	None	1
	Not sure	2
	Do not know	1
	8 h	1
	2 days	2
	6 days	1
	No specific time allocated	2
Disaster-related activities/functions during WIL	Report writing and assessments	2
	Practical exposure and site visits	3
	Project-based learning	1
	Unspecified or variable tasks	1
	No clear requirement/uncertain	3
Industries or work areas involved in disaster WIL	Municipal authorities	5
	Community engagement initiatives	2
	Unspecified or unclear	3
Is assessment required during WIL?	Yes	6
	No	2
	Do not know	2
Is the WIL assessment graded?	Yes	6
	No	2
	Do not know	2
Who conducts WIL assessment?	Academic institution	5
	Jointly by academic and industry	4
	I am not sure	1
Assessment weighting in disaster management module	None	3
	10%	1
	20%	1
	25%	1
	Uncertain	4

WIL, work-integrated learning.

It is a bit surprising to note that some participants were not aware whether there are WIL days allocated for disaster management, while others indicated that their institutions do not provide WIL opportunities for disaster management. Across institutions, different types of activities or functions were described as part of WIL, including site visits, report writing and project-based learning. Most of the institutions reported that students are mostly placed in municipal authorities for WIL experience, with one participant specified in the Directorate of Disaster Risk Unit within the municipality. Two participants indicated doing WIL as service-learning or community engagement. In addition, six participants noted that WIL for disaster management is assessed, although only 3 participants provided varied (10%, 20% and 25%) assessment weightings.

### Disaster management training and environmental health needs

While reflecting on the alignment of the disaster management module to the needs of the EH profession, on a rating of poorly, very poorly, well, very well and neutral, four participants thought the curriculum aligned with the profession’s needs very well, four participants also thought it aligned well, while two participants were neutral. Consequent to the above perceptions, all participants revealed a strong reliance on industry consultations and academic research to ensure that disaster management module content stays current and relevant to the needs of the EH profession. Encouragingly, four participants cited the use of students’ feedback, outlining the importance of students’ contributions in shaping the course materials. Regarding the changing landscape of EH, most of the participants (six out of ten participants) indicated that they update their module content annually to integrate new developments and best practices of disaster management. Three participants indicated that they generally wait for the curriculum review, which is updated every two years, while the other two participants reported a prolonged review cycle of every five years. Of course, these review cycles may be influenced by different institutional policies and the level of rapidness to respond to the changing, emerging trends of disaster-causing risks.

The responses to the question: ‘Do you feel that the course structures provide adequate coverage of global disaster management practices?’, indicate divided views among participants. Out of ten participants, six answered ‘Yes’, while the other four respondents answered ‘No’, indicating that a significant portion of participants perceived some gaps in the curriculum. The responses to open-ended questions revealed several systemic, teaching and resource-based challenges faced by academics and experts involved in teaching, namely:

- limited capacity-building opportunities for lecturers
- curriculum design and content-related challenges
- insufficient practical exposure and WIL integration
- resource constraints.

## Discussion

This study evaluated the training content, course structures and competency frameworks currently utilised to prepare future EHPs in South Africa for disaster management roles. The findings drawn from the experiences and perceptions of disaster management experts affiliated with accredited institutions offering EH training revealed both strengths and notable gaps in the disaster management module within the EH degree programme. Since the national recurriculation of the EH qualification from a diploma to a degree in 2016, disaster management has been formally introduced as a core module to enhance the competencies of EHPs in anticipating, responding to and managing disasters (Mbola et al. 2024).

Considering that disaster management remains a relatively new focus area within the EH profession, the small number of study participants with specialised expertise was not surprising. This trend corresponds to findings in other health professions, where formal training and academic capacity in disaster management remain limited and underdeveloped (Achora & Kamanyire 2016; Berhanu et al. 2016; Farah, Pavlova & Groot 2023; Naser & Saleem 2018; Olu et al. 2018). Even though all experts permanently involved in delivering the disaster management module held at least a master’s degree (NQF Level 9) and had valuable experience in EH practice, several reported lacking formal pedagogical or technical training specific to disaster management. This has implications for their confidence and effectiveness in teaching the subject. Existing literature emphasises the importance of institutional support through staff development and targeted capacity-building programmes for lecturers introducing new subjects such as disaster management, especially in health-related disciplines, where the pace of curricular change is rapid (Fernandez & Audétat 2019; Salajegheh et al. 2020, 2024). Noteworthy initiatives led by the National Disaster Management Centre (NDMC) in collaboration with South African universities such as North-West University, the University of the Free State, and others have begun to address this gap. These partnerships aim to enhance disaster management education and professional development (NDMC 2025; North-West University 2025; University of Free State 2025). At least two participants in this study reported having engaged in short training programmes offered through these collaborative efforts.

### Module structure and design

Findings revealed inconsistencies in the duration and structure of disaster management modules across accredited institutions, with some offering it as a six-month semester course while others extended it over a full academic year. This variation underlines a broader issue of non-standardised curriculum implementation, identified by participants as a significant challenge. Specifically, discrepancies in credit weighting and allocated instructional time were found to be misaligned with the depth and breadth of content expected in the disaster management module. Such misalignment constraints between credit standards and actual instructional time can compromise the attainment of expected learning outcomes, specifically in content-heavy modules like disaster management (Maphosa, Mudzielwana & Netshifhefhe 2014). Standardisation could ensure comparability of learning experience across the institutions and further ensure that EH graduates acquire the core competencies required for the practice. As Shyr et al. (2021) and Srigyan and Fortun (2025) have emphasised, disaster education requires not only theoretical grounding but also pedagogical methods that are interactive and context-sensitive to be effective.

The contrasts in module duration, structure and enrolment figures across institutions further highlight a lack of standardisation in the delivery of disaster management education within EH programmes. This inconsistency may influence the depth and scope of competencies acquired by students, which is particularly concerning given the growing demand for EHPs to respond effectively to disaster situations (Mbola et al. 2024; Ning et al. 2014; Reischl et al. 2008; Shah 2022). The absence of defined prerequisite modules in several institutions may further affect the integration of disaster management content within the broader curriculum, potentially limiting students’ ability to synthesise prior knowledge when dealing with complex emergencies (Koen et al. 2024; Panda et al. 2020; Rofiah, Kawai & Hayati 2021).

### Core content, competency areas and teaching approaches

Concerning disaster management content and competencies, most institutions were found to comprehensively prioritise foundational elements essential for understanding the broader context and operational systems of disaster management (see Table 2). Accordingly, competencies such as risk identification, hazard prioritisation and mitigation planning are adequately addressed, reflecting a strong theoretical and practical emphasis in current training (The African Academy for Environmental Health 2010; Wisner & Adams 2002). This alignment is consistent with the core responsibilities of EHPs, particularly in hazard identification and risk mitigation (Eldridge & Tenkate 2008; Gamboa-Maldonado et al. 2012; Rodrigues et al. 2021; Ryan et al. 2017). However, a notable gap exists in the area of leadership and inter-sectoral coordination competencies, especially in the context of leading disaster response teams. This finding is concerning given that the literature identifies leadership limitations as a persistent challenge among practising EHPs, limiting their capacity to effectively collaborate with multidisciplinary disaster response stakeholders (Dhesi 2018; Fos, Honoré & Honoré 2021; Mbola et al. 2024; Rodrigues et al. 2021). Without adequate preparation in leadership roles, emerging EHPs may be unable to fulfil critical responsibilities during disaster response operations.

Regarding teaching approaches, the study found that while most institutions rely heavily on traditional face-to-face instructional modes, methods such as case studies, group work and guest lectures were frequently reported. Despite the recognised value of innovative and practical teaching methods in disaster management education, approaches such as service-learning, community engagement and simulation exercises remain underutilised in many EH programmes (Mbola et al. 2024). This limitation may hinder students’ ability to translate theoretical knowledge into practice, especially in real-world conditions that require rapid decision-making and effective team coordination. Simulation exercises have been shown to enhance students’ confidence and preparedness for disaster scenarios. For instance, Cowling, Swartzberg and Groenewald (2021) found that emergency medicine trainees reported increased confidence following simulation-based training, even if their theoretical knowledge did not significantly change. Likewise, Fifolt et al. (2023) demonstrated that interprofessional disaster simulations improved students’ understanding of incident command systems and coordination of services, highlighting the effectiveness of experiential learning in complex emergency contexts. Furthermore, Kunguma and Mapingure (2023) also demonstrated how integrating community-based projects into disaster management curricula enabled students to apply theoretical concepts to the actual community challenges, thus enhancing their practical skills and community awareness.

### Practical learning and work-integrated learning

In addition to the underutilisation of experiential methods, the study revealed a limited integration of digital tools in disaster management teaching. Most participants (*n* = 9) reported minimal use of technological platforms, with only one institution indicating meaningful digital integration. This shortfall is concerning, as digital competencies such as real-time data management, GIS mapping, mobile surveillance and virtual simulations are increasingly crucial in modern disaster preparedness and response (Jose & Dufrene 2014; Tasantab et al. 2023). Without these skills, EH graduates may be inadequately prepared for modern disaster environments, where digital tools play a critical role in coordination, early warning systems and situational analysis. Reliance on the regular lecture-based approaches alone may limit the students from benefiting from the true complexities of disaster management practice and its real-life dynamics (Kunguma & Mapingure 2023; Tasantab et al. 2023).

The study findings also noted an ambiguity regarding the requisite duration and structure of WIL specific to disaster management. Participants highlighted difficulties in securing WIL placements within municipal disaster management directorates, often because of logistical barriers or a lack of structured collaboration frameworks. This omission has resulted in inconsistencies in WIL implementation across institutions, with some students receiving limited or no exposure to disaster response activities during their training.

While WIL is a core component of the EH curriculum in South Africa, requiring students to complete a minimum of one hundred days of supervised practical training, the exclusion of disaster management as a formally recognised function within the EHPs’ scope of practice contributes to fragmented and inconsistent exposure (Cape Peninsula University of Technology 2019; Mbola et al. 2024; South Africa 2009). This situation not only restricts access to relevant experiential learning but also reflects a gap in the guidelines that govern WIL placements in the EH profession. Participants emphasised the need for more dedicated time within disaster-related placements, suggesting that students should spend at least two to three days embedded in disaster management centres or departments to gain hands-on experience in emergency planning, coordination and response. This recommendation aligns with the literature, which highlights that structured experiential learning opportunities such as immersive simulations and field exposure enhance the ability of students to apply theoretical knowledge under pressure, foster decision-making and build interprofessional collaboration skills (Cowling et al. 2021; Fifolt et al. 2023). The underutilisation of disaster simulations and other immersive teaching methods, such as project-based learning and community engagement, hinders the development of core competencies necessary for disaster preparedness and response (Guo et al. 2025). These findings align with global educational trends, which emphasise the importance of authentic learning environments in preparing future health professionals for crises (Mbola et al. 2024; Mutasa & Coetzee 2019; Rijumol, Thangarajathi & Ananthasayanam 2010; Suckale et al. 2018; Wells et al. 2013).

### Study recommendations

**FIGURE 2:**
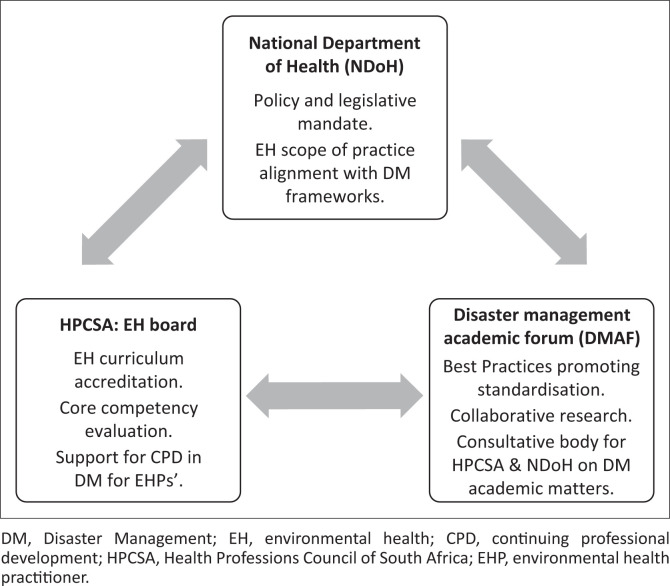
Environmental health disaster management standardisation and key contributing stakeholders.

These findings underline the critical need for a coherent national framework to guide the design, implementation and assessment of disaster management training in EH programmes. An alignment in the curriculum with the existing national disaster risk reduction strategies, such as the South African Disaster Management Framework (South Africa, 2003), professional competency standards outlined in the EH scope of practice (South Africa 2009) and the African Academy for EH content (The African Academy for Environmental Health 2010), could support more consistent and contextually relevant training for EH students. Furthermore, the integration of core public health competencies and experiential learning aligned with the Sendai Framework for Disaster Risk Reduction (United Nations Office for Disaster Risk Reduction 2015) would enhance preparedness and response capacity among EHPs. Standardising module duration and entry requirements across institutions could ensure that graduates are uniformly equipped to meet the challenges of disaster risk management across varied and vulnerable South African settings. These improvements can be accomplished through the proposed framework, which highlights the roles of key stakeholders involved in or influencing EH professional training and practice (shown in Figure 2).

#### National Department of Health

The NDoH is responsible for developing policies that regulate EH practice, including disaster management. One of its key responsibilities is to define and regularly update the scope of practice for EHPs to ensure alignment with evolving public health challenges, including disaster preparedness and response (Mbola, Human & Melariri 2019; South Africa 2004, 2009). Regardless of this mandate, the current EHPs’ scope of practice remains insufficiently aligned with the *Disaster Management Act (Act No. 57 of 2002)* and international frameworks such as the Sendai Framework for Disaster Risk Reduction (South Africa 2002; United Nations Office for Disaster Risk Reduction 2015). This misalignment emphasises the urgent need to integrate disaster management competencies and align with the Sendai Framework’s priority areas, especially those focusing on strengthening disaster risk governance and enhancing disaster preparedness for effective response, which would improve national readiness and ensure that environmental health training is standardised across academic institutions (United Nations Office for Disaster Risk Reduction 2015). This alignment can be achieved through collaboration with the HPCSA’s EH Professional Board, which oversees the accreditation, regulation and curriculum standards for environmental health qualifications (HPCSA 2019; Mbola et al. 2024; National Department of Health 2014).

#### Health Professions Council of South Africa: Environmental Health Board

The HPCSA conducts regular audits of EH training institutions to ensure compliance with minimum standards (HPCSA 2019). However, current inconsistencies in how disaster management is integrated into WIL highlight systemic gaps that emanate from the limited scope definition. Addressing these inconsistencies by integrating disaster-related competencies into the core curriculum would promote uniformity and ensure that graduates are adequately prepared to engage in disaster risk management roles. Additionally, higher education institutions play a central role in equipping EH students with the relevant competencies outlined in the scope of practice and must therefore ensure that the disaster management curriculum is consistently and effectively implemented across programmes (Department of Higher Education and Training 2012).

#### Disaster management academic forum

To facilitate standardisation across institutions, the formation of a Disaster Management Academics Forum (DMAF) is recommended. Bajow et al. (2022) suggest that forums of this nature may ensure quality and uniformity of the education curriculum, facilitate international cooperation and enable evaluation and continuous improvement. This forum would bring together academics involved in teaching disaster management within the seven South African HEIs offering EH degrees. The DMAF would enable collaborative curriculum development, promote best practices and provide a platform for joint research initiatives. For instance, the EmTASK course exemplifies how collaborative academic efforts can lead to innovative training programmes that address the complexities of disaster risk reduction (Righi et al. 2021). Additionally, it could serve as a capacity-building mechanism for academic staff responsible for delivering disaster management content, ultimately enhancing the national competency base in disaster risk reduction (Ferman 2002).

### Future research

Given the exploratory nature of this study and the small sample size (*N* = 10), future research should replicate this investigation with a larger sample of EHPs and disaster management experts across South Africa. Conducting such a study, possibly five years from now, could enable a retrospective assessment of how disaster management training evolves from the first cohort of the new EH degree. Future research could also include comparative analyses across other countries and fields of EH to benchmark curricula, identify best practices and evaluate the impact of standardised national modules on developing core competencies in disaster risk reduction, emergency response and recovery.

## Conclusion

This study has shown the evolving landscape of disaster management education with EH degree programmes in South Africa. Although the inclusion of disaster management as a core module since the national recurriculation in 2016 marks a significant advancement, the findings reveal systemic inconsistencies and critical gaps in curriculum implementation. Key challenges identified included the lack of standardisation in module duration and structure, inadequate integration of work-integrated learning and digital learning methodologies, and insufficient alignment between academic training, the EHPs’ scope of practice and national disaster risk reduction frameworks. Despite the foundational knowledge and competencies currently embedded in EH curricula, such as hazard identification and risk mitigation, it is expected that future EHPs may be underprepared in areas critical to effective disaster response, including leadership, coordination and the use of modern technological tools, which is currently visible in the field. For EH graduates to fulfil their potential role as frontline workers in disaster preparedness and response, training must go beyond theoretical training. It must include immersive, context-sensitive and technologically relevant learning experiences.

To address the identified gaps, a clear national curriculum that aligns disaster management training with the South African Disaster Management Framework, the EH scope of practice and the Sendai Framework for Disaster Risk Reduction is needed. Furthermore, the establishment of a DMAF is proposed to ensure curriculum standardisation, academic collaboration and continuous quality improvement across institutions.
